# Model for home-preschool continuity in linguistically and culturally diverse settings

**DOI:** 10.3389/fpsyg.2024.1408452

**Published:** 2025-01-29

**Authors:** Mila Schwartz, Hanna Ragnarsdóttir

**Affiliations:** ^1^Oranim Academic College, Kiryat Tiv'on, Israel; ^2^Faculty of Education and Pedagogy, University of Iceland, Reykjavik, Iceland

**Keywords:** family language policy, language education policy in ECEC, home-preschool continuity, linguistically and culturally diverse children, linguistically and culturally responsive teaching, family funds of knowledge

## Abstract

With the advent of international freedom of movement, we are witnessing a rapid influx of children from diverse linguistic and cultural backgrounds in mainstream preschools. Preschool education scholars have argued that teachers must work collaboratively with these children’s families to support their “linguistic security” and well-being. The paper presents a conceptual model integrating linguistically and culturally responsive teaching with family funds of knowledge, language education, and family language policies. It highlights the interaction between these constructs that may lead to home-preschool continuity. The model is firmly grounded in three theoretical perspectives: Bronfenbrenner’s ecological model, which emphasizes the importance of the environment in a child’s development; Epstein’s model of parental involvement, which highlights the various ways parents can be involved in their child’s education; and Schwartz’s concept of agency in interactions between teachers and parents, which underscores the importance of mutual understanding and collaboration between these two agents. The model has the potential to guide research focusing on parents’ and teachers’ agency in enacting language policy and addressing cultural values. With its transformative potential, this model opens horizons for practical solutions for the interaction between these agents.

## 1 Introduction

This paper frames home-preschool continuity^1^ construction from sociolinguistic perspectives in linguistically and culturally diverse contexts. It offers an integrated model connecting such constructs as linguistically and culturally responsive teaching (hereafter LCRT), family language policy (hereafter FLP), language education policy, and family funds of knowledge. This model explains how these constructs are related to home-preschool continuity (hereafter HPC). The paper analyzes (1) how parents view their communication with teachers and cope with and respond to their pedagogical approaches and language education policy; (2) how teachers regard or disregard FLP through their perceptions, beliefs, and practical steps toward HPC.

This analysis’s starting point is to claim that fruitful relationships between family and preschool are possible in cases where teachers and parents, as agents, listen and respond to each other’s voices (e.g., Ragnarsdóttir, 2021a; Schwartz, 2022; Tobin, 2019). Thus, the paper aims to answer how continuity could be realized in the face of challenges teachers face in classrooms with linguistically and culturally diverse children (hereafter LCDC) who come from immigrant families speaking language/s other than the socially dominant one at home and who maintain the cultural heritage of the country of origin. These children can also be defined as bi/multilingual since they learn a novel and usually socially dominant language in preschool and are exposed to one or more languages in their home environment.

Concerning the analyzed studies, the paper does not consider itself a thorough, comprehensive overview of the existing research on HPC. Since this research domain is dynamic and growing, we focus on recent studies on how families’ efforts to maintain their home language^2^ and culture interact with teachers’ language education policy and pedagogical approaches supporting these efforts. Appendix 1 briefly describes the selected studies.

Regarding methodological approaches, the reviewed studies are mainly ethnography-oriented. These studies draw on qualitative research methodologies involving classroom observations and in-depth interviews with preschool teachers and parents. Although ethnographic research does not permit statistical generalization, it brings the emic perspectives of parents and teachers as “the insider’s or, as anthropologists call it, the informant’s view of reality” (Morey and Luthans, 1984, p. 29). Thus, ethnography as a research method permits insights into how parents perceive communication with teachers, how teachers understand their role in building HPC, and how they relate to families’ cultural and linguistic backgrounds. In the following sections, we will present the conceptual model of HPC.

## 2 Conceptual model

**Figure 1:**
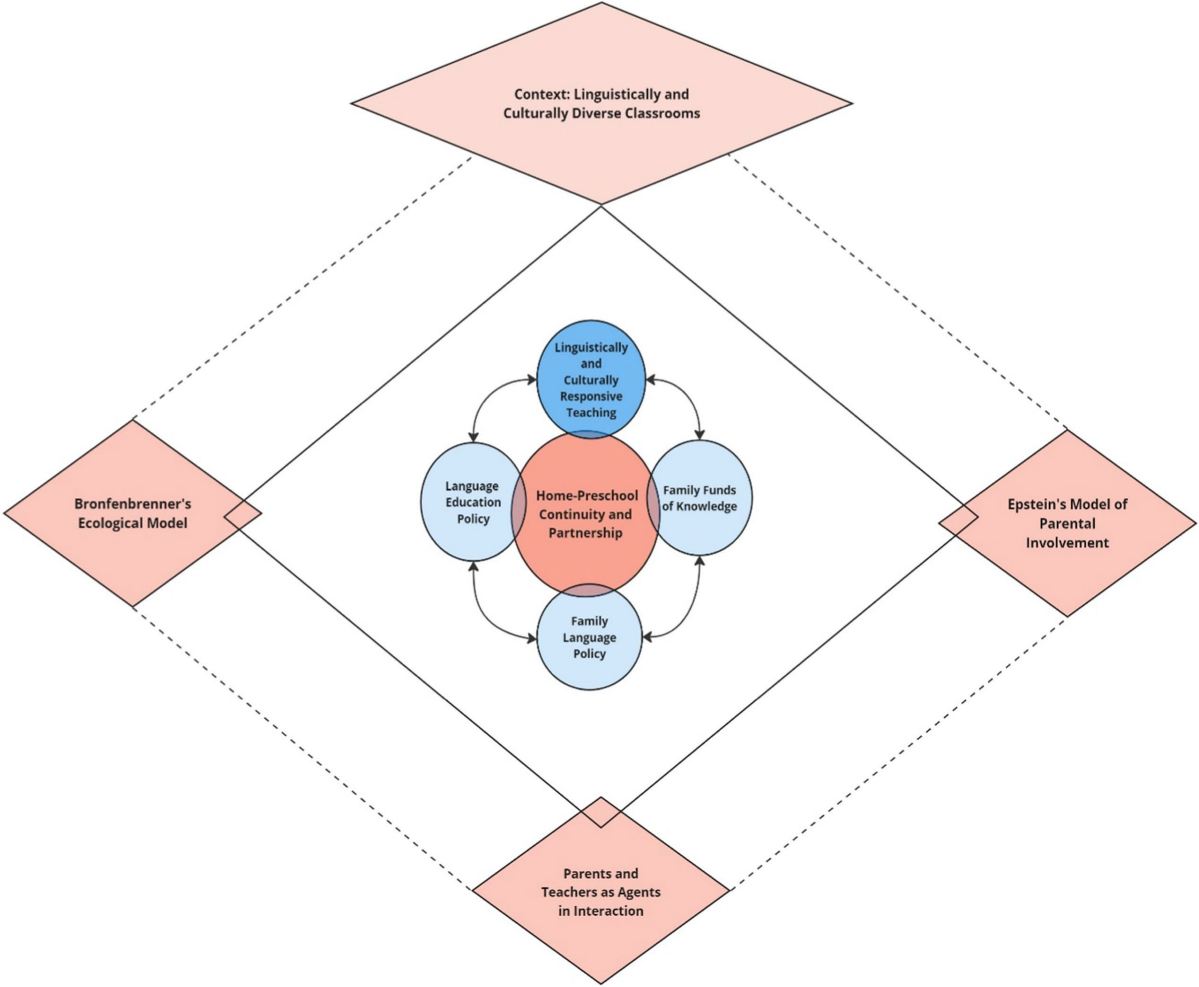
Model for home-preschool continuity in linguistically and culturally diverse settings.

During the last two decades, there has been an increasing body of data on FLP and classroom language policy and practice but as *separate concepts* (e.g., Curdt-Christiansen, 2018; Nandi, 2018; Palviainen and Curdt-Christiansen, 2022; Schwartz and Verschik, 2013). However, as noted by Curdt-Christiansen (2018, p. 422), “tightly knit families do not live in a vacuum, isolated from the larger sociocultural environment” such as educational institutions. Nevertheless, the interaction between FLP and language education policy in the early education context has just recently drawn scholars’ attention (e.g., Bezcioğlu-Göktolga, 2022; Nandi, 2018; Schwartz, 2024). Moreover, a connection between FLP and family funds of knowledge with preschool teachers’ pedagogy, such as LCRT, has not yet been discussed. By claiming that preschool and home create a continuum connecting these two spheres of a child’s initial life experience, we propose a conceptual model in Figure 1 connecting the four constructs: LCRT as a pedagogical approach, language education policy, FLP, and family finds of knowledge.

To knit the proposed model with the underlying theory, we will start with a brief presentation of three fundamental theories: Bronfenbrenner’s (1979) ecological model of child development, HPC in light of Epstein’s (2001) model of parental involvement, and the concept of teachers and parents as agents in interaction elaborated by Schwartz (2018, 2022, 2024). After that, to situate the model, we will address and connect its four constructs. This presentation will be illustrated by selected examples from recent studies demonstrating how these constructs are tied. Finally, future directions in research resulting from the proposed model will be outlined.

## 3 Fundamental concepts of the proposed model

### 3.1 Bronfenbrenner’s ecological model

Bronfenbrenner’s ecological model provides a thorough framework for comprehending interactions between a child and the ecology of his or her development. This theory offers a method to investigate the role of socio-cultural and linguistic interactions by applying five significant systems—micro, meso, exo, macro, and chronosystems. This paper will refer to four systems: micro, meso, exo, and macrosystems, explained below.

According to Bronfenbrenner (1994), daily interactions at home and in the classroom, between parents and children, between teachers and children, and between children constitute a microsystem where the most significant developmental processes occur. Parents’ beliefs about how children learn language(s) and their role in this process may significantly impact children’s experience of language learning and their beliefs about it. This role of the family was theorized within the concept of FLP, discussed below. A mesosystem related to interactions between caregivers, parents, and preschool teachers is of primary interest in this paper. Specifically, at the meso level, the teacher and parents, as agents in interaction, need to be aware of each other’s preferences regarding the child’s development and education. An exosystem “refers to one or more settings that do not involve the developing person [child] as an active participant, but in which events occur that affect, or are affected by, what happens in the setting containing the developing person” (Bronfenbrenner, 1979, p. 25). Regarding the scope of this paper, language education and family policies in preschool classrooms might be influenced by events without a child’s presence, such as parents’ engagement in social networks, including an ethnolinguistic community (e.g., Nandi, 2018). The macrosystem constitutes a more extensive network of cultural beliefs, societal values, political trends, and “community happenings” (Swick and Williams, 2006, p. 372). This article addresses macrosystems in the context of state and ethnolinguistic community language policies that may influence language education and family policies.

### 3.2 Epstein’s model of parental involvement

Epstein’s (2001) model of parental involvement explores school, family, and community partnerships. Although this model was elaborated for the school context, it is certainly relevant to the preschool context and our discussion about home-preschool continuity. Epstein (2001) notes that there is “an endless variety of characteristics and situations of students, families, schools, and communities” (p. 4) that need to be taken into account. Therefore, educators need to understand the different contexts in which these families and children live. This also applies to early childhood educators. Epstein (2001) also emphasizes that without understanding the different contexts of families, teachers work alone and not in partnership with other important people in children’s lives.

The family and school relations model accounts for various changes, including “history, development, and changing experiences of parents, teachers, and students” (Epstein, 2001, p. 27). Her model comprises overlapping or non-overlapping spheres representing the family, school, and community. She explains that the degree of overlap is controlled by three forces: “Time, experience in families, and experience in schools” (p. 27). The internal structure of the model, on the other hand, includes “interpersonal relationships and influence patterns of primary importance” (p. 30).

Later, Epstein’s (2011) work on school, family, and community partnerships emphasized that there are multiple strategies and methods for establishing and maintaining communication with diverse families. She stresses the importance of appreciating the diversity of each family, including family cultures, histories, values, religions, and talents. This includes developing and implementing activities in partnerships between schools and families that build on families’ strengths and backgrounds. Such activities will help students, families, and educators understand and appreciate similarities and differences in cultural layers and life experiences (Epstein, 2011).

### 3.3 Teachers and parents as agents in interaction

From the point of view of social psychology and education, Biesta and Tedder (2007) view agency as a critical idea in modern educational theory and practice, which was recognized as early as the Enlightenment period. The scholars add that agents always act not only in an environment but “*by means* of an environment,” that is, the agency is a result of “the interplay of individual efforts, available resources, and contextual and structural ‘factors’ as they come together in particular and, in a sense, always unique situations” (Biesta and Tedder, 2007; p. 137).

Epstein’s and Bronfenbrenner’s models view teachers and parents as agents in interaction to provide favorable conditions for the child’s early development and well-being. Drawing on this idea, Schwartz (2018, 2022, 2024) elaborated an ecological approach to children’s early language experiences, stressing the critical role of how primary caregivers interact as agents at the mesolevel of a child’s development. Relying on this claim, the researcher called on scholars to explore how these agents engage in dialogue and work together to support children’s bilingual or multilingual growth by maintaining open communication, exchanging insights, knowledge, and materials, and fostering an encouraging language learning environment. She argued that teachers and parents bring their beliefs and values into interaction as grounds for the agency because people will not act unless they believe they have the power and capabilities to produce results (Bandura, 1997). This interaction could be built on personal backgrounds and life experiences that may activate teachers’ and parents’ agency enactment.

To recap, the theories discussed above pave the way to viewing HPC in the context of linguistically and culturally diverse classrooms as a precondition of a child’s well-being and “linguistic security” (Bergeron-Morin et al., 2023, p. 29). They highlight the role of family and teachers as agents in interaction who can negotiate their language policies and cultural practices to advance HPC. The following section will explore how the proposed model refers to the interaction between these agents and their language ideologies.

## 4 Constructs of the model and connections between them

This section will define four contracts building the discussed model and show how these interrelated constructs may foster continuity between home and preschool environments.

### 4.1 Linguistically and culturally responsive teaching (LCRT)

This conceptual paper asserts that teachers implementing LCRT as a pedagogical approach can promote continuity between home and preschool. The target pedagogical concept appears in various sources in different forms (discussion of them is not within the scope of our paper). What is essential is that Ηοllie (2012), for the first time, coined the term culturally and linguistically responsive pedagogy by emphasizing “the language aspect of the culture” and defining it as:

Culturally and Linguistically Responsive Pedagogy (CLR) is the validation and affirmation of the home (indigenous) culture and home language for building and bridging the student to success *[sic]* in the culture of academic and mainstream society (Ηοllie, 2012, p. 23).

She argues that this pedagogical approach addresses children’s cultural and linguistic needs. By adding “linguistic” to the previously accepted notion of “responsive teaching,” Ηοllie (2012) emphasizes that “our language is a representation of our heritage, including family, community, and history” (p. 19). This point aligns with Vygotsky (1978) explanation of the connection between language and culture, claiming that language is one of the cultural tools that mediate cognitive development. Thus, language may be viewed as shaping and being shaped by cultural contexts and as a part of these contexts. Similarly, the proposed model views language and culture as intertwined concepts and connects the linguistic and cultural aspects of a child’s early development and education. By connecting language and culture, LCRT pedagogy underscores the importance of creating a ‘safe space’ (Conteh and Brock, 2011) in classrooms where young children and parents can communicate in their home languages and appreciate the value of maintaining their home cultures.

In recent years, LCRT has grown to promote teaching practices emphasizing reciprocity, respect, and a deep understanding of classroom linguistic and cultural differences, primarily within Western European and North American contexts. It also recognizes home languages and cultures as assets (e.g., Arvanitis, 2018; Hollie, 2012). Teachers aim to “create a caring, respectful classroom climate that values students’ cultures,” deliver meaningful and relevant instruction to children’s life experiences, and cultivate trusting partnerships with families (Perso, 2012; p. 66). This connects us to family funds of knowledge as a cornerstone concept of the LCRT and one of the constructs of the proposed model, which will be explained in the following section.

Addressing the children’s linguistic needs by LCRT pedagogy can be exemplified by implementing a language mediation strategy. For instance, in a study by Eliyahu-Levi and Ganz-Meishar (2019), the researchers analyzed various forms of language mediation that create a ‘safe space’ for African immigrant families to communicate with preschool teachers in Israel. For example, it has been observed that teachers conveyed messages to parents who were not proficient in Hebrew, the socially dominant language, using pantomime, illustration, personal examples, and body.

On the other hand, mainstream teachers’ underestimation of home language and culture maintenance may have serious consequences:

…dual strategy of exclusion and condemnation of one’s language and culture, fostering disdain for what one knows and who one is, has another critical consequence regarding schooling. It influences children’s attitudes towards their knowledge and personal competence. That is, it creates a social distance between themselves and the world of school knowledge (Moll, 2001; p. 13).

#### 4.1.1 Family funds of knowledge

As LCRT adopts an asset view of families, this perception is detailed by a more accurate presentation of customs, traditions, experiences, and language policy, namely family funds of knowledge. Family funds of knowledge are “historically accumulated and culturally developed bodies of knowledge and skills essential for household or individual functioning and well-being” (Moll et al., 1992, p. 133). This concept provides “a new way of thinking about the knowledge that comes from the experiences of immigrants by valuing them as resources for teaching and learning” (McDevitt, 2021, p. 126). In this way, drawing on families’ funds of knowledge enriches the classroom and empowers them as experts in their language policy and cultural values. Moreover, incorporating family funds of knowledge into the classroom curriculum promotes a respectful attitude toward daily home linguistic and cultural practices.

Recent research by Ragnarsdóttir (2021a) shows that teachers can establish HPC by using family funds of knowledge to welcome the reception of immigrant children entering preschool. In this study, teachers and children in Iceland were prepared to welcome Syrian refugee classmates. Specifically, teachers thought in advance about how to prepare peers for the arrival of new classmates. The children sang an Arabic song, which their music teacher had translated into Arabic and taught to the children in Arabic. As noted by the preschool principal, this welcoming reception seems to have played a significant role in the child’s smooth socialization and progress in Icelandic.

In addition, teachers may learn about family funds of knowledge through home visits. In a study by Whyte and Karabon (2016), teachers in the USA participated in a professional development program and conducted home visits of the chosen focal child’s family. The traditional target of home visits shifted from informing the parents about child learning to learning and gathering “information from the families” (Whyte and Karabon, 2016; p. 208). In this way, as Whyte and Karabon (2016) claim, teachers as active agents may encourage the family’s engagement in their child’s education. The researchers also asserted that by entering children’s homes, the teachers play the two-fold role of teacher and ethnographic researcher “to act mutually as an insider and an outsider, as a learner and a teacher” (Whyte and Karabon, 2016, p. 209).

As family funds of knowledge are an integrated part of LCRT, teachers can include them in classroom curricula (e.g., Melzi et al., 2019; Schwartz and Dror, 2024). Indeed, a recent study by Schwartz and Dror (2024) focused on how ECEC teachers created a continuity between home and preschool among 3-4-year-old children from the Bnei Menashe immigrant community^3^. As reported by the teacher, the parents “were very enthusiastic and were most happy about having a place [in preschool]” and expressed a feeling of belonging to the preschool community (Schwartz and Dror, submitted, p. 23). This feeling was created by incorporating the families’ funds of knowledge within the daily program by reading self-made bilingual Hebrew (L2)-Mizu (L1) books during preschool time and encouraging the parents to take the books home. The teacher believed reading these books at home could promote parent–child interaction during quality time and stimulate both parents and their children’s progress in Hebrew as a novel language. In addition to progress in Hebrew, the teacher believed that bilingual books could support the children’s home language maintenance. She engaged the parents to cooperate with her in the bilingual book reading. The feeling of belonging was enhanced by integrating into curriculum topics related to the target community’s cultural traditions (food, clothes) and learning about the geography of northeastern Indian territory, the community’s homeland (Schwartz and Dror, 2024). There were also interactive display walls with common words and greetings in Hebrew (L2) and Mizu (L1), with transliteration of Mizu into Hebrew letters, to facilitate smooth communication with Bnei Menashe children and their families.

To conclude, as Epstein (2001) asserted, parents might feel empowered when preschool teachers create welcoming outreach programs. By implementing LCRT, including a reference to family funds of knowledge, teachers may connect the child’s experience with the home language and culture and family intimacy with the classroom atmosphere to advance HPC.

### 4.2 Language policy

This paper asserts that LCRT as pedagogy is intertwined with language education and family language policies as concepts of the discussed model. In turn, the paper claims that the outcomes of these interactions *influence* the continuity between home and preschool, which is within our scope. The connections are complex and non-linear and reflect the broader sociolinguistic context in which interactions between home and preschool occur. The following subsections will define language education and family language policies and bring research illustrating how these constructs may promote HPC in interaction with LCRT.

#### 4.2.1 Language education policy

Language policy has been defined in several ways. According to Kaplan and Baldauf (1997), “language policy is a body of ideas, laws, regulations, rules, and practices intended to achieve the planned language change in societies, groups, or systems” (p. xi). Nandi (2024) highlights that language policy is a “complex interplay between individuals’ actions and policy-making actions on the national/regional or local levels, always involving some form of engagement, mediation, and persuasion among diverse agents who act as policy arbitrators in situations where two or more languages are being used” (p. 5).

Language education is “a kind of language management” (Spolsky, 2017, p. 2). It is generally built on explicit or implicit language education policies concerned with language practice questions in educational settings (Shohamy, 2008; Spolsky, 2017). Specifically, language education policy encompasses various aspects, including the language of instruction, bilingual/multilingual education, language rights, and home language acknowledgment. Teachers may enact their agency in language education policy, for instance, by implementing LCRT.

In the context of our paper, language education policy concerning preschool children’s home languages is under the scope. This policy implemented in ECEC settings may encompass planning, practices, and ideologies related to the teaching and learning of languages (Palviainen and Curdt-Christiansen, 2022). This policy plays a vital role in shaping young children’s multilingual or monolingual development regarding maintaining their home language and acquiring socially dominant languages (Bergeron-Morin et al., 2023). Additionally, language education policy may influence monolingual children’s receptiveness to different languages in ECEC, representing an initial step in fostering plurilingual skills that are crucial from a lifelong learning perspective and raising language awareness (e.g., European Commission, 2011; Lourenço, 2024).

At the classroom level, language education policy is influenced by language ideology on the macro state or national level (Shohamy, 2006). Generally, in many Western countries, the involvement and cooperation of immigrant parents in decision-making are cornerstones of national ECEC curricular guidelines (e.g., Bergeron-Morin et al., 2023). In the context of our paper, there has recently been a growing tendency to include language orientations in ECEC policy documents and teacher education guidelines in many Western countries (e.g., Alstad and Sopanen, 2020; Bergroth and Hansell, 2020; Schwartz et al., 2022). For example, the Ministry of Education and Culture of Finland (2017) outlines Finland’s plan to “become a multilingual and multicultural country,” including early foreign language learning as well as support for heritage languages (pp. 12–13). This development is inevitably linked to teachers’ increasing awareness of the need to involve all parents in classroom activities (Bergroth and Hansell, 2020).

#### 4.2.2 Family language policy

For many linguistically and culturally diverse families, the ECEC institution becomes the first place to negotiate between their home language policy and the institution’s language education policy (Bergeron-Morin et al., 2023). Parents may feel insecure about their FLP and children’s bi/multilingual upbringing (Van der Wildt et al., 2023). This insecurity may be related to the pressure of competing demands, namely, the desire to pass on their home language(s) intergenerationally to their children while providing them the best opportunities to learn the socially dominant language (e.g., Okita, 2002; Schwartz, 2010). In these cases, ECEC practitioners must engage with parents (Bergeron-Morin et al., 2023; Van der Wildt et al., 2023).

Fishman (1991), an early proponent of proactive language maintenance at home and in the community, proposed a model for reversing language shift. He claimed that the family acts as a natural boundary, a bulwark against outside pressures. Indeed, advocacy of intimacy and privacy may help family members maintain their home language and prevent its substitution by the socially dominant language. This is because family context is a critical initial stage in children’s language socialization and is their closest language ecology.

Similarly, this role of the family was conceptualized within the notion of FLP, which, according to King et al. (2008), “provides an integrated overview of research on how languages are managed, learned and negotiated within families” (p. 907). In parallel, Kopeliovich (2006) and Schwartz (2008) called for the adaptation of Spolsky’s (2004, 2009) language policy model to the family level. Spolsky (2004) distinguished between three interconnected components in the language policy of a speech community: “Its language practices – the habitual pattern of selecting among the varieties that make up its linguistic repertoire; its language beliefs or ideology – the beliefs about language and language use; and any specific efforts to modify or influence that practice by any kind of language intervention, planning or management” (Spolsky, 2004, p. 5). Spolsky argued that language policy at the family level might be analyzed concerning language ideology, practice, and management, as in any other social unit.

### 4.3 Patterns of interaction between language education and family language policies

Preschool education provides children’s first formal exposure to language learning experiences beyond the home. High-quality teacher-child interactions in ECEC environments may foster young children’s language development (Vernon-Feagans et al., 2013; Walker et al., 2020). However, mainstream teachers often lack awareness of the family’s efforts to maintain the home language while supporting children’s acquisition of the socially dominant language (Bergeron-Morin et al., 2023; Schwartz, 2024). In these circumstances, vital questions arise when classroom teachers seek to understand children from diverse cultural and linguistic backgrounds: How are children’s languages and cultures supported at home? How do home language practices differ from classroom experiences? Moreover, how do preschool teachers support LCDC’s language development in interaction with their families?

Ideally, home and preschool should maintain continuity in language policies to create a sense of security among young children regarding their language home language use and development and socially dominant language learning. This was evidenced by Bergroth and Palviainen (2016), focusing on Swedish-Finnish-speaking bilingual classrooms in Finland. However, as discussed below, in preschools, contingent upon mainstream monolingual education, teacher-parent interaction in children’s linguistic development *may* or *may* not lead to continuity. Based on our model, it depends mainly on the *nature* of the interaction between language education and family language policies. From this point of view, we identified five interactional patterns: (1) Tension between language education and family language policies; (2) A lack of specific language education policy and uncertainness regarding FLP; (3) Teachers’ intentional implementation of language education policy supporting home languages; (4) FLP as a *Happylingual approach*; (5). Home-preschool partnership. As will be addressed below, most of these patterns of interaction are mediated by teachers’ implementation of LCRT.

#### 4.3.1 Tension between language education and family language policies

In a case where LCRT does not underlay classroom pedagogy, there is growing evidence of how language education policy ignores FLP, leading to tension between preschool and home language ideologies. For example, in a study examining the interaction between language education and family language policies among members of the Turkish immigrant community in the Netherlands, Bezcioğlu-Göktolga and Yağmur (2018) conducted observations and interviews with Turkish families and Dutch mainstream teachers working with four-year-old children. Although Turkish parents expressed reliance on teachers’ knowledge and professionalism, the research revealed tensions regarding teachers’ influence on their FLP.

The study underscored the complex dynamics involving parental aspirations, educational advice, and language practices. Specifically, the Turkish parents demonstrated bilingual orientations and a strong desire for their children to receive quality education to ensure future success. They were open to educators’ recommendations, even if it meant adjusting their language practices at home. For instance, upon teachers’ suggestions to increase Dutch language exposure, parents engaged their children in more Dutch-oriented activities such as watching Dutch television programs and hiring tutors to enhance their Dutch skills. However, significant conflict arose over the use of language. While teachers supported the use of Turkish until children reached the age of four (when compulsory preschool education began), they advocated prioritizing Dutch and reducing Turkish input after that. This recommendation conflicted with parents’ aspirations to maintain their home language alongside acquiring Dutch. This disparity in language ideologies and practices between parents and teachers underscores a significant challenge in promoting children’s bilingualism. The lack of collaboration between teachers and families highlights mainstream teachers’ difficulties in addressing FLP because of their adherence to the monolingual language education policy.

#### 4.3.2 A lack of specific language education policy and uncertainness regarding family language policy (FLP)

Families may face various challenges regarding supporting their children’s bi/multilingual development and education. In addition to their efforts to provide their children with a rich language/s learning environment in the home context, they need to be supported by educators through, for example, by teachers who consult parents. However, as noted above, research has indicated that teachers may be uncertain regarding the advice they are occasionally supposed to provide parents about bi/multilingual upbringing at home and FLP (Bergeron-Morin et al., 2023; Van der Wildt et al., 2023).

Moreover, it may be that FLP is rarely discussed during parent-teacher meetings. Thus, for example, Van der Wildt et al.’s (2023) recent quantitative study conducted in Flandres with a substantial sample of multilingual language minority parents explored whether parents and teachers discuss language upbringing in an advisory talk. It was found that 67% of the total respondents have not received or asked any advice or discussed any linguistic upbringing of their children. In a case where the teachers and parents did discuss the children’s linguistic upbringing, the teacher’s most frequent recommendation was to speak the language parents know best with their young children. This was followed by suggesting that one parent speaks one language and the other speaks another. Fortunately, more parents were given multilingual rather than monolingual advice, promising to sustain young children’s bi/multilingual development and home language maintenance.

#### 4.3.3 Teachers’ intentional implementation of language education policy supporting home languages

Based on the principles of LCRT, mainstream teachers may also *intentionally* implement language education policies that encourage immigrant parents to invest efforts in home language maintenance, as evidenced in the study of Chinese parents community in Australia by Hu et al. (2014). In this study, the teachers were aware of the value of home language maintenance for a child’s development. Therefore, they respected children’s right to speak their home language in preschool and actively advocated this right to parents with different views on their children’s linguistic development. The socio-linguistic context of this study involved Chinese parents’ FLP with a preference for their children to speak English over their home language. This preference is driven by the belief that proficiency in English is crucial for academic success and future career opportunities in an English-dominant society. Parents assumed that speaking English would help their children integrate better into the broader community.

At the same time, the teachers considered that the children using the home language in the early childhood center is beneficial “in terms of children’s social development, confidence and feelings of belonging” (Hu et al., 2014; p. 262). This view of empowering children through students’ linguistic and cultural capital in everyday learning aligns with LCRT (Perso, 2012). The teachers mainly reported promoting bilingualism by incorporating the home language in classroom activities and creating an inclusive environment that values linguistic diversity. To resolve parents’ concerns about children’s competence in English, most teachers used parent-teacher meetings, newsletters, and other forms of communication to explain the benefits of bilingualism and align educational practices with parental aspirations. They actively convinced parents that the children have sufficient exposure to English through interactions with staff and English-speaking peers. To conclude, the study underscored the need to negotiate language education and family policies and foster collaborative relationships between teachers and parents to support HPC.

As addressed above, recent changes in national childhood curricula of some Western countries focus on the linguistic needs of LCDC at the micro level of classroom practices and provisions for home languages (e.g., Bergroth and Hansell, 2020; Dražnik et al., 2022). This tendency may activate teachers’ agency in supporting home languages and cultures by applying LCRT. For example, Sweden’s state-national approach to language education has led to a preschool curriculum incorporating a progressive language education policy empowering FLP within mainstream monolingual classroom settings. Within these reforms, Puskás and Björk-Willén (2017) explored the implementation of modified Swedish-speaking curricula, which introduced bilingual teachers and activities in children’s home languages (e.g., conducting story time in Romani).

#### 4.3.4 Family language policy (FLP) as a Happylingual approach

Learning a socially dominant language as a novel language is a “long drawn-out process” (albeit daily input) (De Houwer, 2009, p. 95) demanding both educational and parental engagement (Schwartz, 2022, 2024). Further, De Houwer (2020) asserts that children who grow up in a linguistically diverse environment need not only to develop skills in their home language but also acquire skills in both their home language and the socially dominant language for their *harmonious development*. The harmonious development means parents’ positive attitude towards both languages in the child’s ecology. This leads us to the *Happylingual approach* towards childhood bilingualism, coined by Kopeliovich (2013), which means that parents must color children’s environmental language in cheerful colors. They should express “unbiased attitude to diverse languages that enter the household and [show] respect for the language preferences of the children” (Kopeliovich, 2013, p. 51). This approach indirectly connects FLP with language education policy in mainstream preschool classrooms, putting child agency at the center (Kopeliovich, 2013; Soler and Zabrodskaja, 2017).

Although immigrant parents may be eager to promote their child’s harmonious bilingual/multilingual development, teachers must be aware that, in many cases, they cannot support the socially dominant language at home because of their low competence (Norheim and Moser, 2020). In this case, they should relate to this issue sensitively and empathetically and suggest creative solutions such as communication with peers who are native speakers and the use of technology and media (e.g., Norheim and Moser, 2020; Schwartz, 2024).

#### 4.3.5 Home-preschool partnership

Drawing on Epstein’s (2001) model of parental involvement, a continuity between home and preschool regarding language policies and family funds of knowledge may also be identified as a home-preschool partnership. Family engagement in classroom life can be facilitated through open and trusting communication and relationships between teachers and parents as key children’s primary caregivers (e.g., Ragnarsdóttir, 2021a).

Existing, albeit limited, data indicate that teachers and parents can collaborate if they are aware of and attentive to the values of language education and family language policies, and funds of knowledge (e.g., Hu et al., 2014; Mary and Young, 2017; Norheim et al., 2023; Ragnarsdóttir, 2021a,b,c; Tobin et al., 2013). To illustrate, a recent large-scale quantitative research project provided data about teachers’ perceptions of partnership with parents in linguistically and culturally diverse classrooms in four European countries: England, Italy, Norway, and the Netherlands (Norheim et al., 2023). This project showed, among others, the positive relationship between teachers’ self-reported multicultural practices drawn on family funds of knowledge and their views of partnerships. Specifically, more tremendous implications of multicultural practices were significantly related to such partnership aspects as stronger shared beliefs with parents (i.g., similar views on a child’s behavior) and reciprocal relations with them (i.g., welcoming parents initiatives) (Norheim et al., 2023; p. 20).

Another qualitative study by Lastikka and Lipponen (2016) focused on immigrant parents’ perspectives on partnership with ECEC teachers in Finland. As noted above, the Finnish language education policy supports children’s home languages and cultures and aims to respect them. The 13 interviewed immigrant parents came from diverse backgrounds, and their children were engaged in a mainstream daycare center in Helsinki. The parents reported about teachers’ practices aligning with the LCRT principles. For example, they highlighted that the daycare acknowledges family funds of knowledge by presenting diverse religious practices and developing respectful attitudes toward them among the children. As one father noted “children were not obliged to attend Christmas parties or attend church, and dietary restrictions were accommodated” (Lastikka and Lipponen, 2016; p. 8). In addition, the parents remarked that the greetings were written in different languages, and songs were sung in these languages. FLP was addressed by organizing language clubs with exposure to home languages. The children were encouraged to speak their mother tongue at home. The researchers concluded that “creating a cooperative partnership between educators and immigrant families helps them engage in open dialogue and establish a mutually respectful and shared understanding of children’s development” (Lastikka and Lipponen, 2016; p. 88).

Another example of an emergent home-preschool partnership was explored by Ragnarsdóttir (2021a). A starting point for changes in current Islandic policies regarding multicultural and multilingual issues in education, stressing that “knowledge of more than one language is a treasure that must be nurtured and developed, as all languages open up the doors to different cultures and make our lives richer” (Icelandic Ministry of Education, Science and Culture, 2020; p. 4). The researcher focused on six monolingual preschools in three different municipalities in Iceland. She investigated how principals and teachers partner up with immigrant refugee families. The beginning of the partnership was observed as the parents were interested in collaboration with teachers and utilized the ideas that they had suggested. This was illustrated by giving an example of ‘communication books’; these books comprised pictures of the refugee family and the preschool staff, and their names were included. Children used to bring these books home to develop their content and then return the books to the preschool. These books also incorporated words in Icelandic to support the acquisition of Icelandic as a socially dominant language.

The studies discussed above show how teachers and parents, as agents, perceive their communication and negotiate language education and family policies, and classroom cultural activities. They highlight that the partnership can be promoted by balancing respecting the family’s wishes with the educational benefits of maintaining the home language and supporting multilingual development. A critical point that the data reveals is that there was a tendency for one-way, teacher-laden relationships in advancing partnership. Thus, in most cases, families were not part of active engagement in decision-making.

## 5 Conclusion

The model discussed in this paper proposes a comprehensive approach to understanding the continuity between home and preschool by exploring interrelationships between four constructs: LCRT, language education, family policies, and family funds of knowledge. We consider these aspects to be interconnected building blocks rather than isolated components, as they have the potential to develop HPC through their connections.

The theoretical foundations supporting the model bolster its credibility and applicability in early education. Drawing on Bronfenbrenner (1979) meso level of a child’s development, Epstein’s (2001) model of parental involvement, and Schwartz (2024) ecological approach toward early language education, the model underscores that parents and teachers are not isolated actors but agents in collaboration responsible for a child’s linguistic and cultural security. The paper further extended these concepts by identifying five interactional patterns between language education and family language policies, as discussed. It was also addressed that FLP as a private domain can be embedded within exosystem interactions with the language policy of ethnolinguistic communities (Bezcioğlu-Göktolga and Yağmur, 2018; Hu et al., 2014). Finally, it was shown how the macro level, the broader context of state/national language policy, and the current turn towards cultural diversity may directly influence the teachers’ classroom language education policy and practices, raise attention to family funds of knowledge, and therefore advance HPC.

We identified several critical issues connecting theory and existing data underlying the model that should be resolved in future research. First, although there is growing research focusing on the opinions of parents and teachers, only a few studies have explored the perspectives of both agents on establishing relationships. Still, both agents had much to contribute to the dialogue of linguistic and cultural practice and policy at home and in the education setting when they were asked to discuss their concerns. This dialogical communication paved the way for HPC. Another critical point is a lack of focus on children’s agentic perceptions of home-preschool communication. Children as active subjects have experience and voice. Moreover, they do not blindly accept the opinions of caregivers in their nearby orbit regarding their bilingual/multilingual experience but question them and form opinions of their own (e.g., Bergroth and Palviainen, 2017; Schwartz, 2024). Parents and teachers must be highly sensitive to these voices if this is the case.

We also consider the model to have the potential to inform practical strategies for developing HPC. In this way, it aims to empower policy-makers, teachers, and parents to implement it in practices such as collaborative workshops where parents share their funds of knowledge. Additionally, the model encourages caregivers to reflect on their beliefs and practices since, as was illustrated, through such reflections, they can negotiate discrepancies in their perceptions regarding the roles of home and socially dominant languages in preschool and home environments and prevent misunderstandings and tensions stemming from a lack of communication (e.g., Hu et al., 2014).

## Data Availability

The datasets presented in this article are not readily available because privacy. Requests to access the datasets should be directed to milasch@bgu.ac.il.

## Footnotes

^1^In most cases, we used the terms “preschool” or “early childhood education and care” (hereafter ECEC) setting/institution interchangeably to address early childhood education contexts embracing preschool-age children and avoid a multiplicity of notions. Still, we also used other terminology, such as daycare or kindergarten, again to follow the authentic terminology used by the authors of specific publications.

^2^As Schalley and Eisenchlas (2020) suggested, we use the notion of “home language.” Home language “embraces the contexts where language use is negotiated” at the micro level of family members’ communication (p. 2). This notion signifies “the language or languages of the child’s immediate environment outside mainstream education” (Schwartz, 2024, p. 8). In some cases, we apply terms such as minority language and heritage language instead of home language to follow the terminology presented by specific studies.

^3^The Bnei Menashe community is an ethnoreligious group residing primarily in the northeastern Indian states of Manipur and Mizoram. This community claims descent from one of Israel’s lost tribes and practices Judaism. Starting in the 1980s, groups of Bnei Menashe began immigrating to Israel under the Law of Return, which grants them citizenship.
