# Supplementary material for: Model for home-preschool continuity in linguistically and culturally diverse settings
Source: Front Psychol. 2025 Jan 29;15:1408452. doi: 10.3389/fpsyg.2024.1408452 (PMC11813688; doi:10.3389/fpsyg.2024.1408452)
Supplement: Supplementary file 1 [file Table_1.docx]

**Appendix 1: Summary of selected studies with research questions and methods**

| **Author(s) in the alphabetic order** | **Research Questions** | **Methodology/tools** | **Participants** |
| --- | --- | --- | --- |
| Bergroth and Palviainen (2016) | (1) “What major discourses are circulated by the parents and the practitioners when talking about languages and the ECEC partnership?”  (2) “What kind of participatory roles …emerge in these discourses displaying obligations, desires, abilities and competencies for acting on bilingualism?”  (3) “What kind of change in current ECEC partnership practices is needed, if any?” (Bergroth and Palviainen, 2016; p. 4). | Qualitative study was a part of a larger, ethnographic research project.   - Semi-structured interview | 9 Finish-Swedish bilingual families and six practitioners at three Swedish-medium minority language ECEC units in Finland |
| Bergroth and Hansell (2020) | 1. “How can in-service training for pedagogical practitioners be further developed so that it better promotes LA [language awareness] within the whole professional learning community?” 2. “What kind of dynamics of minority–majority language positions can be identified in the audio-recorded practitioner group discussions?” (Bergroth and Hansell, 2020; p. 89). | Action research.   - 9 audio-recorded practitioner group discussions with four to five participants in each group | 41 primary participants and 165 secondary participants on sites (i.e., each primary participant’s respective professional learning communities) working in mainstream monolingual ECEC settings in Finland |
| Bezcioğlu-Göktolga and Yağmur (2018a) | 1. “What is the agency of teachers in FLP, home language use, and the role of language(s) in learning and school achievement?” 2. “What are the parents’ aspirations for teachers’ opinions on language learning and home language use?” (Bezcioğlu-Göktolga and Yağmur, 2018a; p. 225). | Qualitative study.   - Semi-structured interviews | - 20 second-generation Turkish families (40 parents). - 5 Dutch mainstream teachers working with children from age four. |
| Eliyahu-Levi and Ganz-Meishar (2019) | 1. What actions have been taken by kindergarten teachers to strengthen their personal relationships with African migrant parents? | Qualitative study was based on the phenomenological approach.   - Individual interviews | - Five kindergarten teachers working in five kindergartens in two large cities in Israel. |
| Hu et al. (2014) | 1. How do Australian early childhood educators perceive home language usage in early childhood settings? 2. How do educators perceive Chinese parents’ views about their children’s language use in early child centres? 3. How do educators attempt to negotiate partnerships with Chinese parents when there is a mismatch between their views? | Qualitative study.   - Semi-structured interviews | - Five teachers in Australian ECEC centres. |
| Lastikka and Lipponen (2016) | (1) “What are the most important ECEC practices for the immigrant families?”  (2) “How do these practices help and support the families in their everyday life?” (Lastikka and Lipponen, 2016; p. 77). | Qualitative study.   - Individual interviews | - 13 immigrant parents - one daycare in Finland |
| Norheim et al., (2023) | (1) “How do ECEC professionals view their partnerships with parents in multicultural  classrooms?”  (2) “Do ECEC professionals’ characteristics and practices predict their views of partnerships with parents in multicultural classrooms?” (Norheim et al., 2023; p. 6). | Quantitative study was a part of the research project *Inclusive Education and Social Support to Tackle Inequalities in Society*.   - Questionnaire | - 130 ECEC professionals from four European countries: England, Italy, Norway, and the Netherlands. |
| Puskás and Björk-Willén (2017) | 1. How are language policies implemented in a preschool group where three languages (Swedish, Romani and Arabic) are spoken daily? | Qualitative ethnographic study.   - Field notes - Video-recorded classroom observations - Policy documents collection - Interviews | - One mainstream preschool classroom in Sweden. - Three teachers: one classroom teacher two first language teachers teaching Arabic and Romani, respectively. |
| Ragnarsdóttir (2021a) | 1. What are the experiences of parents, principals, and teachers in the reception and the first year of the children’s schooling? | Qualitative study.   - Semi-structured interviews | - 11 refugee parents (6 families) - five principals - four heads of divisions - three educators   The participants worked in 6 preschools in three Icelandic municipalities. |
| Schwartz and Dror (2024) | 1. What are teachers’ beliefs about LEP in their classroom? 2. Why do teachers apply LED [language education policy] in their classroom? 3. How do teachers model their FoK/I [funds of knowledge and identity] to young children as part of their LCRT? 4. How do teachers address the children’s FoK [funds of knowledge] in their classroom? (Schwartz and Dror, 2024; p. 5). | Ethnographically orientated case study.   - Classroom observations - Language policy documents - Photographs of the classroom language landscape - Language resources provided by the teaching staff. - Semi-structural interviews. | - One teacher and three teacher assistants come from diverse linguistic and cultural backgrounds. - One mainstream preschool “with children aged 3–4 years, 50% of the classroom population belonged to the Bnei Menashe community whose home language was Mizu (one of the Indian languages)” (Schwartz and Dror, 2024; p. 4). |
| Van Der Wildt et al. (2023) | 1. “What are MLM multilingual language minority] [parents’ perceptions regarding their communication with and trust in the caregivers within Flemish childcare?” 2. “What are MLM [multilingual language minority] parents’ language expectations towards the caregivers within Flemish childcare?” 3. “What are MLM [multilingual language minority] parents’ perceptions regarding the caregivers’ language practices that might provide language continuity between home and facility within Flemish childcare?” 4. “Do MLM [multilingual language minority] parents receive linguistic advice in a multilingual upbringing from the caregivers within the Flemish childcare?” 5. “What linguistic advice do MLM parents receive from the caregivers within the Flemish childcare?” (Van Der Wildt et al., 2023; p. 5). | Quantitative study.   - Questionnaire | - 404 LM [language minority] parents with children aged two months to three years old. - 85 childcare facilities in Flanders. |
| Whyte and Karabon (2016) | “What happened when early childhood teachers entered homes to learn from families and identify their FoK [funds of knowledge]?” (Whyte and Karabon, 2016; p. 207). | Qualitative study.   - Home Visit Reflections: The teachers were asked to complete three home visits with their chosen focal child’s family each year. The participants individually wrote about their experiences in Home Visit Reflections. | 15 public prekindergarten teachers in the USA participated in a professional development program with a focus on “developmentally and culturally responsive mathematics teaching.”  (Whyte and Karabon, 2016; p. 211). |
